# People’s Willingness to Vaccinate Against COVID-19 Despite Their Safety Concerns: Twitter Poll Analysis

**DOI:** 10.2196/28973

**Published:** 2021-04-29

**Authors:** Fabian Eibensteiner, Valentin Ritschl, Faisal A Nawaz, Sajjad S Fazel, Christos Tsagkaris, Stefan Tino Kulnik, Rik Crutzen, Elisabeth Klager, Sabine Völkl-Kernstock, Eva Schaden, Maria Kletecka-Pulker, Harald Willschke, Atanas G Atanasov

**Affiliations:** 1 Division of Pediatric Nephrology and Gastroenterology Department of Pediatrics and Adolescent Medicine, Comprehensive Center for Pediatrics Medical University of Vienna Vienna Austria; 2 Ludwig Boltzmann Institute for Digital Health and Patient Safety Medical University of Vienna Vienna Austria; 3 Section for Outcomes Research Center for Medical Statistics, Informatics, and Intelligent Systems Medical University of Vienna Vienna Austria; 4 Ludwig Boltzmann Institute for Arthritis and Rehabilitation Vienna Austria; 5 College of Medicine Mohammed Bin Rashid University of Medicine and Health Sciences Dubai United Arab Emirates; 6 Department of Oncology Cumming School of Medicine University of Calgary Calgary, AB Canada; 7 Faculty of Medicine University of Crete Heraklion Greece; 8 Ludwig Boltzmann Institute for Digital Health and Prevention Salzburg Austria; 9 Department of Health Promotion Care and Public Health Research Institute Maastricht University Maastricht Netherlands; 10 Department of Anaesthesia, Intensive Care Medicine and Pain Medicine Medical University of Vienna Vienna Austria; 11 Institute for Ethics and Law in Medicine University of Vienna Vienna Austria; 12 Institute of Genetics and Animal Biotechnology of the Polish Academy of Sciences Jastrzebiec Poland; 13 Department of Pharmacognosy University of Vienna Vienna Austria

**Keywords:** COVID-19, SARS-CoV-2, vaccine, vaccination, Twitter, survey, vaccination willingness, vaccination hesitancy, coronavirus, vaccine confidence, willingness, hesitancy, social media, safety, concern, public health, opinion, perception

## Abstract

**Background:**

On January 30, 2020, the World Health Organization’s Emergency Committee declared the rapid, worldwide spread of COVID-19 a global health emergency. Since then, tireless efforts have been made to mitigate the spread of the disease and its impact, and these efforts have mostly relied on nonpharmaceutical interventions. By December 2020, the safety and efficacy of the first COVID-19 vaccines were demonstrated. The large social media platform Twitter has been used by medical researchers for the analysis of important public health topics, such as the public’s perception on antibiotic use and misuse and human papillomavirus vaccination. The analysis of Twitter-generated data can be further facilitated by using Twitter’s built-in, anonymous polling tool to gain insight into public health issues and obtain rapid feedback on an international scale. During the fast-paced course of the COVID-19 pandemic, the Twitter polling system has provided a viable method for gaining rapid, large-scale, international public health insights on highly relevant and timely SARS-CoV-2–related topics.

**Objective:**

The purpose of this study was to understand the public’s perception on the safety and acceptance of COVID-19 vaccines in real time by using Twitter polls.

**Methods:**

We developed 2 Twitter polls to explore the public’s views on available COVID-19 vaccines. The surveys were pinned to the Digital Health and Patient Safety Platform Twitter timeline for 1 week in mid-February 2021, and Twitter users and influencers were asked to participate in and retweet the polls to reach the largest possible audience.

**Results:**

The adequacy of COVID-19 vaccine safety (ie, the safety of currently available vaccines; poll 1) was agreed upon by 1579 out of 3439 (45.9%) Twitter users. In contrast, almost as many Twitter users (1434/3439, 41.7%) were unsure about the safety of COVID-19 vaccines. Only 5.2% (179/3439) of Twitter users rated the available COVID-19 vaccines as generally unsafe. Poll 2, which addressed the question of whether users would undergo vaccination, was answered affirmatively by 82.8% (2862/3457) of Twitter users, and only 8% (277/3457) categorically rejected vaccination at the time of polling.

**Conclusions:**

In contrast to the perceived high level of uncertainty about the safety of the available COVID-19 vaccines, we observed an elevated willingness to undergo vaccination among our study sample. Since people's perceptions and views are strongly influenced by social media, the snapshots provided by these media platforms represent a static image of a moving target. Thus, the results of this study need to be followed up by long-term surveys to maintain their validity. This is especially relevant due to the circumstances of the fast-paced pandemic and the need to not miss sudden rises in the incidence of vaccine hesitancy, which may have detrimental effects on the pandemic’s course.

## Introduction

On January 30, 2020, the World Health Organization’s Emergency Committee declared the rapid, worldwide spread of SARS-CoV-2 and COVID-19 a global health emergency [1]. Since then, tireless efforts have been undertaken in order to mitigate disease spread and its impacts on many different areas of public health, which range from the amount of patient and health care personnel to nationwide public health measures that mostly rely on nonpharmaceutical interventions [2-5]. Several of these measures have already been associated with the reduced transmission of COVID-19 in geographic, region-wide studies [6,7]. By December 2020, the first COVID-19 vaccine candidates were proven to be safe and efficacious in protecting against COVID-19 and were approved by regulators [8-11], and more vaccine candidates are still under development [12]. Consequently, medical societies and experts all over the world have advocated that vaccination against COVID-19 should be prioritized for high-risk groups, especially older people and people with underlying chronic medical conditions that place them at an increased risk of severe outcomes resulting from SARS-CoV-2 infection [13,14].

Since the beginning of the COVID-19 pandemic, traditional media coverage and information distribution via social media channels have been shaping public opinions and international public health strategies [15]. Although this may have positive effects on public health attitudes related to mitigation measures, one should also be aware of the detrimental effects of misinformation in media [16-20]. Misinformation should be of special consideration in the context of social media platforms such as Twitter, since false claims regarding COVID-19 appear to propagate faster on such platforms, as demonstrated in a recent study by Shahi et al [21]. However, even before the COVID-19 “infodemic,” the spread of misinformation on social media platforms, on e-commerce platforms (eg, Amazon [22]), and by prominent celebrities in the United States has led to the emergence of an antivaccine movement, which has detrimental effects on national vaccine programs [23]. The resulting increased incidence of vaccination hesitancy is partly responsible for the re-emergence of measles in the United States almost 20 years after its elimination [24].

The potential implications of social media for public health are becoming increasingly clear, and the medical community was using Twitter as a tool for public health research long before the onset of the COVID-19 pandemic. The primary purposes of its use, as reported in previous health-related studies, are content analysis, surveillance, engagement, recruitment, intervention, and network analysis, and most related studies are being published in the areas of public health and infectious diseases [25]. Such Twitter-based analyses involve important public health topics that are often related to the themes of infectious diseases, such as the public’s perception on antibiotic use and misuse or human papillomavirus vaccination [26,27].

Twitter—a social media platform with about 353 million monthly active users [28]—allows registered users (possible for anyone aged over 13 years) to share short, 280-character texts (also known as tweets) with other users. These tweets may be further categorized into different topics to start discussions. This is done by the use of tagging symbols, such as the hashtag symbol (#). Such discussions may be further facilitated by incorporating Twitter’s built-in and anonymous polling tool. This tool has the potential to obtain insight into public health topics and real-time feedback on an international scale [29].

Surveying public attitudes toward COVID-19 vaccination is of high importance, since it might provide a better understanding of the reasons behind vaccine hesitancy and how to better design vaccine awareness strategies. Given the fast-paced dynamic of the COVID-19 pandemic, the Twitter polling tool seems to be a reasonable instrument for gaining immediate, large-scale, international public health insights on SARS-CoV-2–related topics. We therefore used this opportunity to rapidly collect and analyze international public health data on COVID-19 vaccines by using the Twitter polling tool. Through these efforts, we aimed to explore the potential benefits and limitations of using such a highly relevant digital health tool to investigate a highly important and broadly discussed topic.

## Methods

In 2019, the Ludwig Boltzmann Institute for Digital Health and Patient Safety in Vienna, Austria was launched with the major aim of empowering patients and health care professionals with digital tools and promoting innovative research and the development of digital health and patient safety tools. One of these innovations was the initiation of the Digital Health and Patient Safety Platform (DHPSP) [30], which provides subscribers with an overview of recent scientific publications regarding digital health and patient safety.

**Figure 1:**
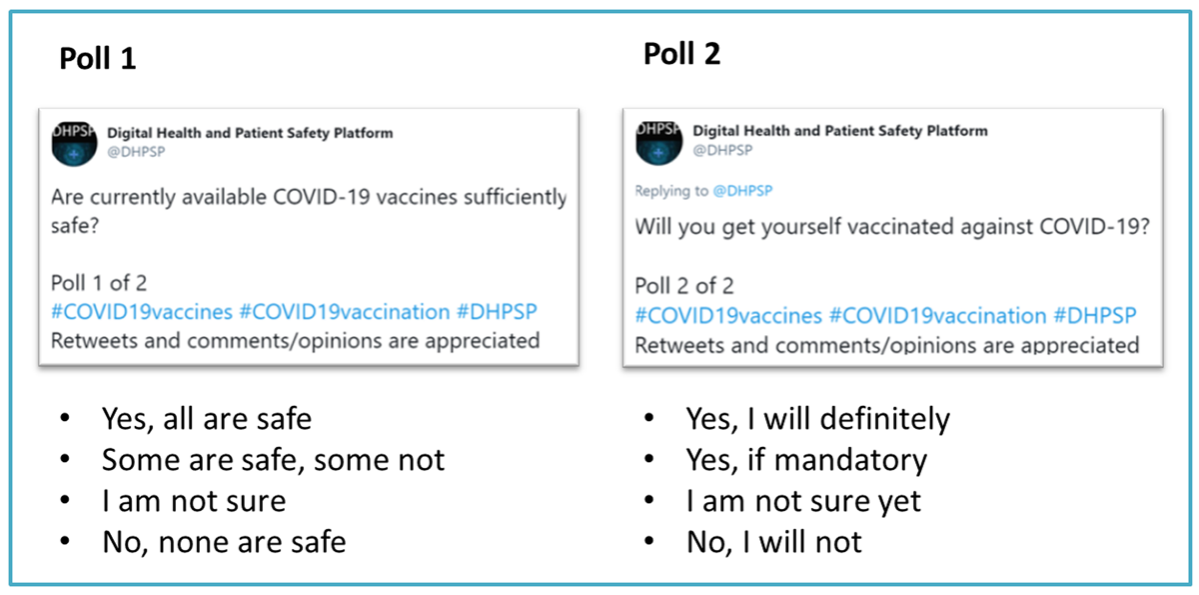
Structure of the two Twitter polls.

For this study, we used the Twitter account of the DHPSP (Twitter handle: @DHPSP) to distribute two polls regarding COVID-19 vaccine safety and acceptance. The polls were developed by the authors and posted on Twitter under the @DHPSP Twitter handle between February 12 and February 19, 2021. Poll 1 addressed the perceived safety of the available (at the time of polling) COVID-19 vaccines (“Are currently available COVID-19 vaccines sufficiently safe?”), whereas poll 2 addressed the confidence or hesitancy of the respondents with regard to undergoing vaccination against COVID-19 (“Will you get yourself vaccinated against COVID-19?”). Both polls were linked (poll 2 was posted as a comment below the tweet for poll 1) and pinned at the top of the DHPSP Twitter timeline during the polling period. The poll questions, which included hashtags for categorization, were limited (by Twitter) to 280 characters. Twitter allows up to 4 answers with a limit of 25 characters (including spaces) for each poll. Therefore, both polls included 4 answers that ranged from total agreement (“Yes, all are safe” and “Yes, I will definitely”) to total disagreement (“No, none are safe” and “No, I will not”) and were presented in the manner of a 4-point response scale. Both polls were categorized using the following hashtags to promote better visibility and facilitate analysis: #COVID19vaccines, #COVID19vaccination, and #DHPSP. Figure 1 displays the detailed construction of both polls on Twitter.

After launching the polls, the first people who could see them on their Twitter timelines were the DHPSP Twitter followers. Twitter poll votes are anonymous and do not allow for the evaluation of respondents’ characteristics (eg, gender). Therefore, in order to at least obtain some data on the characteristics of the audience that was first exposed to the polls, we aimed to analyze the follower characteristics of @DHPSP via the web-based tool Followerwonk [31] on February 20, 2021 (Table 1). In total, the @DHPSP Twitter account had 526 followers at the time of the analysis. Of these, 121 (23%) were male, 69 (13.1%) were female, and 336 (63.9%) did not state their gender on Twitter. A total of 66 (12.6%) @DHPSP followers had more than 5000 followers, 152 (28.9%) had between 500 and 5000 followers, and 308 (58.6%) had less than 499 followers.

**Table 1:** @DHPSP’s Twitter follower characteristics (N=526).

Characteristics		Value, n (%)
**Gender**		
	Male	121 (23)
	Female	68 (13)
	Not stated	337 (64)
**Follower count**		
	<499	305 (58)
	500-5000	153 (29)
	>5000	68 (13)
**Account age (years)**		
	<1	95 (18)
	1-5	158 (30)
	>5	273 (52)
**Language**		
	English	310 (59)
	Spanish	21 (4)
	Other	195 (37)

The polls' body message encouraged people to retweet the polls (“Retweets and comments/opinions are appreciated”; Figure 1), and with each new retweet, the polls gained a bigger audience (consisting of the followers of the retweeting accounts). Moreover, to achieve greater visibility, members and email list subscribers of the DHPSP [30] were asked to support the polls by voting; retweeting the polls; and disseminating them via diverse networking approaches, including direct emails or direct social media messages. The website of the DHPSP and diverse social media accounts of DHPSP members were also used to post hyperlinks to the polls. Additionally, information for the polls was shared through the DHPSP Facebook [32] and LinkedIn [33] accounts.

To characterize the population of users that retweeted the studied polls, we performed a hashtag analysis by using the web-based tool Symplur Signals [34]. We therefore analyzed the number of retweets, users, locations, and languages of all tweets that contained our unique combination of hashtags (#COVID19vaccines, #COVID19vaccination, and #DHPSP) by the end of this study. This was done on the day after the polls were closed (February 20, 2021). To ensure accuracy and to limit interference bias from other Twitter discussions related to our topic, we conducted a Twitter search prior to launching the polls (February 11, 2021), and we confirmed that our combination of hashtags was never used.

Ethical approval was not required for this study, as it was outside the scope of the medical ethics law in Austria. Participation in the studied polls was completely anonymous. Therefore, the collected data were outside the scope of the General Data Protection Regulation [35]. Excluding the poll votes, analyzed parameters such as the number of followers and retweets were based on data that were publicly available on the internet.

## Results

Both of our Twitter polls were pinned to the Twitter timeline of the @DHPSP Twitter account for 7 days, beginning on February 12, 2021. Pinning a tweet permanently places it at the top of a Twitter user's account. Therefore, any new visitors will see this tweet at the top of the visited user's timeline.

Poll 1 (“Are currently available COVID-19 vaccines sufficiently safe?”) received a total of 3439 votes (194,695 views), whereas poll 2 (“Will you get yourself vaccinated against COVID-19?”) received a total of 3457 votes (246,814 views). The analysis of the poll retweets that contained our unique combination of hashtags (#COVID19vaccines, #COVID19vaccination, and #DHPSP) revealed a total of 930 tweets from 375 users. Overall, 262 (69.9%) users posted 1 retweet, 67 (17.9%) users posted 2 retweets, and 46 (12.3%) posted ≥3 retweets. The polls, including all retweets, had a total of 15,446,703 views on Twitter. The top 3 locations of Twitter users who retweeted the polls were the United States of America (n=64, 17.1%), the United Kingdom (n=19, 5.1%), and Canada (n=16, 4.2%). A summary of these details is provided in Table 2. The other top locations (rank 4-10) were India (13 users), Mexico (8 users), Argentina (5 users), Spain (5 users), Australia (4 users), United Arab Emirates (4 users), and Italy (3 users).

**Table 2:** Analysis of poll retweets that contained our unique combination of hashtags (#COVID19vaccines, #COVID19vaccination, and #DHPSP).

Characteristics		Value, n (%)
**Top locations^a,b^**		
	United States of America	64 (17.1)
	United Kingdom	19 (5.1)
	Canada	16 (4.2)
**Number of retweets^b^**		
	1	262 (69.9)
	2^c^	67 (17.9)
	≥3^c^	46 (12.3)
**Top languages^d,e^**		
	English	802 (86.2)
	Spanish	6 (0.6)
	Indonesian	5 (0.5)

^a^Determined based on data derived from the users who indicated their location in their account information on Twitter. During the interpretation of the data, readers should be aware that 50.5% (189/375) of Twitter users did not provide location information on their profiles.

^b^Percentage is based on the number of Twitter users who retweeted the polls (N=375).

^c^Includes regular retweets, retweets with comments, and quote retweets (in which a hyperlink to the original tweet is inserted in a newly composed tweet).

^d^Only the most used languages are indicated. All other tweet languages each accounted for less than 0.5% (5/930) of the retweets.

^e^Percentage is based on the number of retweets (N=930).

In total, 45.9% (1579/3439) of Twitter users who responded to poll 1 (*“*Are currently available COVID-19 vaccines sufficiently safe?*”*) voted with total agreement (“Yes, all are safe”), meaning that the users considered all currently available COVID-19 vaccines to be safe. However, almost as many Twitter users (1434/3439, 41.7%) were not sure about the safety of the available COVID-19 vaccines (voted with “I am not sure”). Interestingly, only 5.2% (179/3439) of the respondents in this poll felt that the available COVID-19 vaccines were generally unsafe (“No, none are safe”). In addition, a total of 7.2% of Twitter users (248/3439) advocated for the safety of some vaccines but felt that not all of them were safe.

Poll 2 explored Twitter users’ confidence or hesitancy toward undergoing vaccination against COVID-19 (“Will you get yourself vaccinated against COVID-19?”). A majority (2862/3457, 82.8%) of the respondents stated, “[y]es, I definitely will [get vaccinated].” In total, 6.8% (235/3457) of Twitter respondents were not yet sure (voted with “I am not sure yet”) about undergoing vaccination, and 8% (277/3457) categorically rejected vaccination at the time of polling (“No, I will not”). Only a minor percentage (80/3457, 2.3%) of Twitter users stated that they would undergo vaccination if it was mandatory.

**Figure 2:**
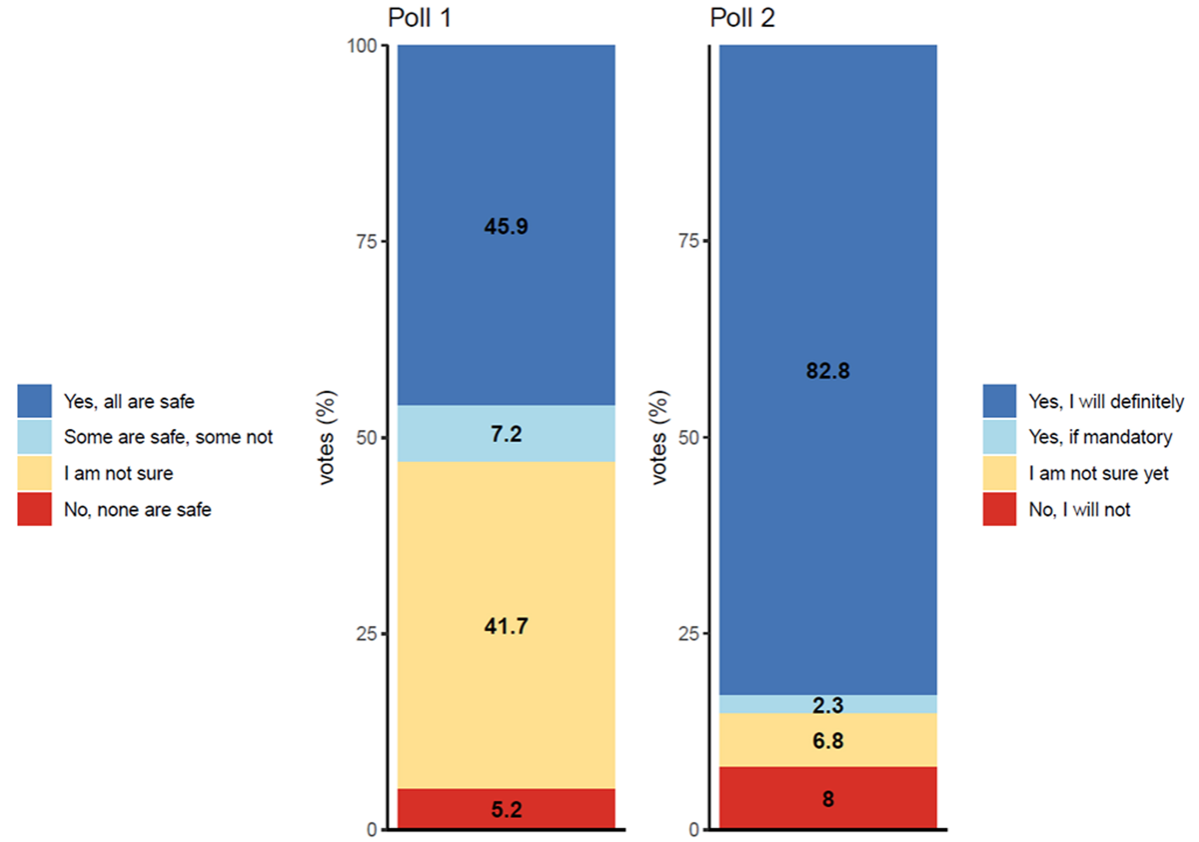
Twitter users' answers to poll 1 (“Are currently available COVID-19 vaccines sufficiently safe?”; respondents: n=3439) and poll 2 (“Will you get yourself vaccinated against COVID-19?”; respondents: n=3457).

A detailed summary of the answers to both polls is provided in Figure 2.

## Discussion

In the context of the fast-paced dynamic of the COVID-19 pandemic, we used the rapid, progressive environment of social media (ie, Twitter) to gain international insights into the public’s opinion on COVID-19 vaccination. We followed a methodological approach that was outlined in a previous study on public attitudes toward telemedicine, which was conducted by Vidal-Alaball et al [29]. They suggested that the Twitter polling tool for quick surveys on timely topics should be used to obtain prompt feedback for new questionnaires before their validation. In this study, by using the DHPSP’s Twitter handle and gaining the support of the retweeting accounts, we were able to validate the Twitter polling approach on a large scale. We obtained a 30-fold higher poll response rate and view rate (impressions) and an 18-fold higher retweet rate compared to those of Vidal-Alaball et al [29]. In the previously mentioned study regarding attitudes toward telemedicine, Vidal-Alaball et al [29] only used the Twitter handle of one of the authors, whereas in our study, an established Twitter network (the DHPSP) was used to achieve a greater reach and higher response rates. Therefore, we were able to not only validate the previously published approach on a larger scale but also demonstrate the definitive advantage of an established user network that is less dependent on single users for achieving a wider reach and higher response rates.

Although this study involves the first scientific Twitter poll analysis of the perceived safety of available (at the time of polling) COVID-19 vaccines and the confidence or hesitancy of respondents with regard to undergoing vaccination against COVID-19, it is not the first survey on this topic in medical literature. In this study, despite the insecurities about the sufficient safety of the available (at the time of polling) COVID-19 vaccines (BNT162b2 by Pfizer-BioNTech, mRNA-1273 by Moderna, ChAdOx1 by AstraZeneca, and Gam-COVID-Vac by the Gamaleya Research Institute of Epidemiology and Microbiology of the Russian Federation), which was observed in 54.1% (1861/3439) of the poll’s respondents, a surprisingly large group of respondents (2863/3457, 82.8%) voted that they would definitely undergo vaccination.

Although this is a positive result, due to the Twitter poll’s anonymous nature, there is no reassurance that the Twitter users who answered the first poll also answered the second poll. In contrast to this study, in a large-scale international analysis of 13,426 participants from 19 countries that was conducted via multiple international, web-based panel providers (Dynata, Opinion Access, Survey Monkey, and Amazon MTurk), Lazarus et al [36] found that only 46.8% of participants agreed to accept a COVID-19 vaccine if it was generally available; 24.7% somewhat agreed; and 14% and 34.1% completely and somewhat agreed to accept a COVID-19 vaccine if it was recommended by their employer, respectively. However, the data from the Lazarus et al [36] study were collected in June 2020. At that time, none of the currently available COVID-19 vaccines were approved by regulatory authorities. Other studies that were conducted during an earlier pandemic phase reported higher rates of willingness to undergo vaccination once a vaccine against COVID-19 became available, ranging from 59% to 75% [37].

Our study may be limited in terms of interpretability. This is due to the fact that the visibility of the polls was widely promoted by the DHPSP, which has a follower base that consists of highly educated individuals with scientific backgrounds or strong interests in science. Since the DHPSP account exclusively posts science-based content, it is reasonable to assume that it attracted followers with interests in science. This assumption is in line with a recent study by Schwarzinger et al [38], which revealed that COVID-19 vaccination hesitancy was highly prevalent among people with low educational levels in the French population. These findings are in line with other, more recent studies that were conducted in France, the United States of America, and Australia [39-41]. As reported and discussed by Kreps et al [42], several factors associated with willingness, hesitancy, advice, and recommendations to vaccinate against COVID-19 are consistent with those in past studies on other vaccines, whereas other factors may be more complex due to the fast-paced dynamic and unpredictable course of the pandemic as well as difficult political and public health actions and communications.

The number of surveys on confidence and hesitancy toward vaccinating against COVID-19 that have been conducted over the course of the pandemic has demonstrated the importance of regular reviews on the public’s opinion toward the effectiveness and safety of vaccines. Such reviews are needed in order to instill public confidence [36]. Lazarus et al [36] also concluded that one of the most important factors for initiating positive health behaviors is credible and culturally informed health communication. With this in mind, health authorities could reach out to the public via rapid, low-barrier, and easy-to-access media platforms, such as Twitter, in order to monitor and instill positive health behaviors through the provision of clear information and credible sources that are tailored to the cultural backgrounds of target populations. As Twitter polls provide the necessary anonymity for confident and large-scale participation and allow for rapid and concise questioning, we propose that this tool is useful for reaching out to the public and addressing public health issues.

The strengths of this tool and this study lie in rapid assessment, the large-scale dissemination of information, and the expeditious retrieval of concise information. The possible strengths of using a preformed network to disseminate information and surveys in order to reach a broad target audience are shown in this study, especially in our comparison of single-user promotion and our promotion method. The very tight restrictions of Twitter polling, including question and answer character limits as well as limits on the total number of answers, might serve as strengths for constraining poll creators to the development of concise and well-formulated surveys, which might result in higher response rates than those of traditional surveys. However, these restraints may also impede the formulation of more complex questions and the clarification of questions and answers. This might interfere with poll results, as a lack of clarity may result in different interpretations among poll participants. Therefore, Twitter polling is better suited for clear, concise, and close-ended questions instead of open-ended and semistructured questions that leave room for interpretation.

**Figure 3:**
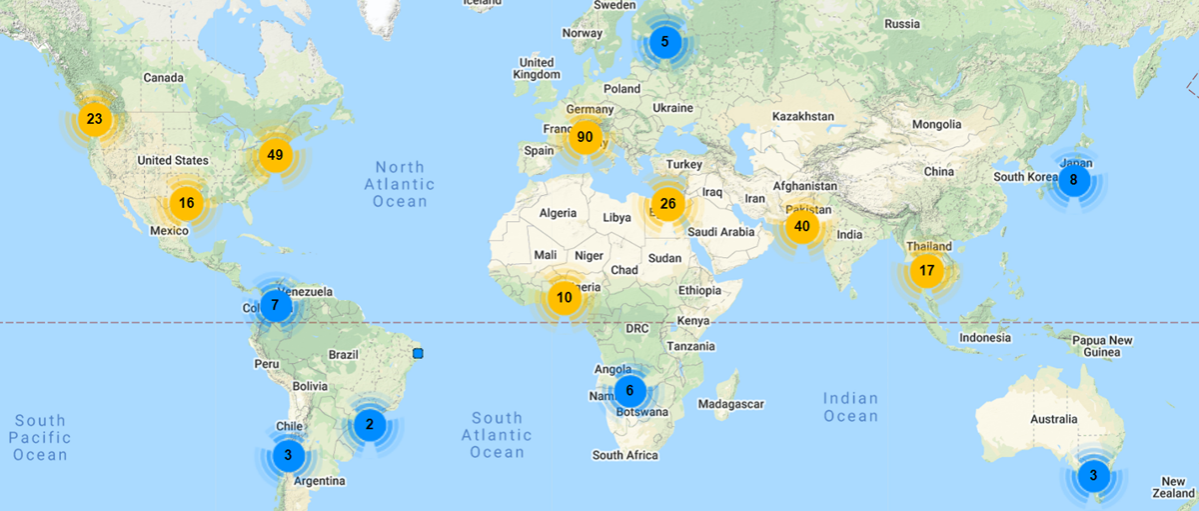
Main locations of the DHPSP's Twitter followers. These data cover only a fraction of the DHPSP followers who indicated their location in their account information on Twitter. DHPSP: Digital Health and Patient Safety Platform.

The pinning and promoting of Twitter polls by specific accounts may also interfere with sample selection and result in biases that may be challenging to mitigate because of the lack of data on the baseline characteristics (eg, gender, age, and socioeconomic status) of participants. However, this challenge can be easily overcome by promoting Twitter polls for a longer period of time across multiple accounts and groups. Nevertheless, it should be noted that complete anonymity can result in the manipulation of votes in the polling tool due to people exploiting multiple usership, as outlined in the study by Vidal-Alaball et al [29]. Even though the analysis of Twitter network followers is made possible by third-party web-based tools, the geographical distribution of the @DHPSP follower network (Figure 3) might not be representative of our polls’ responders, as random Twitter users were able to participate in these polls. The Twitter polls did not undergo a formal validation process. Future studies (eg, those that pilot polling tools) should address the validation of the questions used in this study. However, we were able to gain a better perspective of the users who responded to the polls by using a novel approach, which involved a unique combination of hashtags and a hashtag analysis, to gain insights on the population of users who retweeted the Twitter polls.

In conclusion, despite the high levels of uncertainty regarding the safety of available COVID-19 vaccines in the study sample, the respondents had a high willingness to undergo vaccination. The public’s perceptions and views on health issues are strongly influenced by social media. This underscores the importance of using social media polling tools to understand public health perspectives in real time. Such information can be used to inform public health messaging and communication efforts. Regular surveys on public health issues that use social media platforms may aid in the early discovery of sudden rises in the incidence of COVID-19 vaccination hesitancy among the public before the detrimental effects of the pandemic can manifest.

## Abbreviations

DHPSP: Digital Health and Patient Safety Platform
